# Acute toxicity of herbicide (glyphosate) in *Clarias gariepinus* juveniles

**DOI:** 10.1016/j.toxrep.2016.05.004

**Published:** 2016-05-06

**Authors:** Ali Sani, Muhammad Khadija Idris

**Affiliations:** Department of Biological Sciences, Bayero University Kano, P.M.B 3011, Nigeria

**Keywords:** Toxiciity, Bioassay, Herbicide, Concentration

## Abstract

The constant discharge of agricultural waste into aquatic environment has led to accumulation of heavy chemicals and other variety of pollutants. Herbicides present in these wastes are washed down, carried by rains and flood to nearby aquatic environment. Glyphosate is one of the most popular herbicides used by farmers in Kano because of its active reaction on killing weeds without affecting the crops. A toxicity test of glyphosate was conducted using concentrations of 0, 0.004, 0.005, 0.006, 0.007 ml/l. The mortality rate of each concentration was determined and the physicochemical parameters (Dissolved oxygen and pH) were also determined. The result showed that high mortality occurs at 0.007 ml/l and less mortality was found at 0.004 ml/l. Hence, mortality is dose dependent. DO and pH decreases with increase in glyphosate concentration. Furthermore, the juveniles showed abnormal behaviour. The LC_50_ value at 96 h was 0.0072 ml/l. There was significant difference between the initial and final pH value (*p *< 0.05). On the other hand, the initial and final DO values showed no significant difference (*p *> 0.05). However, correlation between DO and pH showed no significant difference (*p* > 0.05). The findings of this study established that glyphosate has some level of toxicity on *Clarias gariepinus* juveniles. In addition, it was found that mortality, changes in behaviour, DO and pH are dose dependent. Therefore, it was suggested that an appropriate concentration that will not be detrimental to non-target organisms should be used by farmers. Alternatively, Biological method should be used as a substitute for chemical method of controlling weeds.

## Highlights

• Mortality profile of glyphosate (herbicide) on *Clarias gariepinus* juveniles.
• Changes in physicochemical parameters of water after dosing with different concentrations.
• Monitoring of changes in behavioural patterns.

## 1 Introduction

Herbicide is an agent that has the ability to cause death to plants. They are chemicals that are used for agricultural and industrial purposes. They are effective against humans, plants and aquatic organisms such as fish because of their toxicity [1], [2]. Herbicides can be found in large amount in the environment including soil, aquatic and biotic environments. Many herbicides are toxic to fish at very minimal concentration. Several herbicides are recognized for causing renal and hepatic lesions in fish [3]. Therefore, the mass killing of fishes can be attributed to pesticides and herbicides [1], [3], [4]. Similarly, the effects of herbicides are based on

(i) Solubility of herbicides.
(ii) Magnification of herbicides concentration upon entry into the food chain and.
(iii) Persistence of herbicides and transformation to other harmful metabolites upon entry into soil, water and biota [3].

The application of herbicides is reported to have a negative effect on aquatic organisms such as fish. Previous studies describe the wide application of glyphosate as a threat to aquatic environment due to negligence, non-adherence to instructions, lack of knowledge about the negative implication of herbicides and laws that govern the use of herbicides.

Glyphosate is one of the herbicide used for controlling annual and perennial grasses, broad-based leafed weeds, trees and other species [5]. It is one of the established herbicide used worldwide and it is a major pollutant of rivers and surface water. Furthermore, it is perhaps the most important herbicide ever develop because of its low persistence. Some surfactants that are present in the formulation of glyphosate are toxic to aquatic organisms and hence are not suitable for aquatic use [6].

The constant flow of agricultural waste into aquatic environment leads to accumulation of heavy chemicals and other variety of pollutant. Herbicides present in these wastes are washed and carried away by rains and flood to nearby aquatic environment which as a result affect non target aquatic organisms such as fish that serves as a good source of protein. Glyphosate is one of the most popular herbicides used by farmers in Kano because of its effectiveness in killing weeds without affecting the crops.

Fish is considered as a model organism in conducting experimental studies such as toxicological and some pharmacological studies. The potentiality of application of the findings from these researches on humans and other environmental health issues has made fish a more attractive model organism in toxicology research [7].

Glyphosate is considered a probable human carcinogen based on the scientific based evaluation of cancer reported in humans and other laboratory animals [8]. This prompts the need to investigate the toxicity of glyphosate herbicide on *Clarias gariepinus* juveniles through; the determination of mortality profile of glyphosate on *Clarias gariepinus* juveniles; determination of changes in physciochemical parameters at various concentrations and; and observation of changes in the behaviour of *Clarias gariepinus* at various concentrations Valuable data generated would give additional information to the potentials of glyphosate to cause adverse effects on vertebrates (humans inclusive).

## 2 Materials and methods

### 2.1 Collection of test organisms

*Clarias gariepinus* juveniles were collected from a local dealer at BUK road opposite BUK old site. They were obtained from Alimisho Local Government Area of Lagos State, Nigeria, in October 2015. Standard procedure for handling the test organism has been followed. Although, no any institutional animal care and use committee is available in Kano.

### 2.2 Collection of test chemical

Test chemical was collected from shop no M157 Sabon Gari Market, Kano, Nigeria in October 2015.

### 2.3 Preparation of stock solution concentration

Using syringe, 0.1 ml of the test chemical was diluted with 9.9 ml water which gives 10 ml of solution. From the solution, 0.7, 0.8, 0.9 and 1.0 ml were taken to represent 0.07, 0.08, 0.09 and 0.1 ml stock concentration respectively. The concentrations were then converted to ml/l.

### 2.4 Bioassay

Juveniles of *Clarias gariepinus* mean weight 9.0 ± 0.6 g and 10.0 ± 0.5 cm of length collected from Alimisho Local Government Area of Lagos State were used for the study.

Four different glyphosate test solutions were prepared. Each concentration was replicated twice including the control. Four specimens of test organisms (*Clarias gariepinus*) were kept in 25 l plastic tank filled with 15 l borehole water from BUK old campus.

The fish were acclimatized for 24 h. During the acclimation period, fish were not fed and there was no mortality during the period.

Each plastic tank was observed daily for a period of 96 h. Observations were at intervals of 6 h on daily basis. pH and dissolved oxygen (DO) concentration of each plastic tank were also taken on daily basis. Test organisms were not fed during acclimatization of 24 h and during the test period so as to minimize the accumulation of faecal materials. Each experimental unit were observed closely for potential abnormal swimming behaviour and mortality. Death was indicated by less response to stimulus and when the fish turned upside down and sank to the bottom of the plastic tank or when their tail showed no form of movement even when prodded with a glass rod [9].

### 2.5 Statistical analysis

A statistical software named Sigmastat (version 3.5) 2015 was utilized in computing *t*-test between the initial and final pH values, and also between initial and final DO values. The software was also utilized in computing correlation between the pH and DO.

## 3 Results

The result of the mortality rate is shown in Table 1 which shows no mortality in control experiment throughout 96 h. However, less mortality was found in 0.004 ml/l and highest mortality in 0.007 ml/l. This finding indicated that mortality is dose dependent; that is it increases with increase in concentration of glyphosate herbicide.

**Table 1:** Mortality rate of *Clarias gariepinus* juveniles exposed to different concentrations of glyphosate.

Concentration (ml/l)	Initial No.	Final No.	% Mortality
0.000	4	0	0
0.004	4	1	25
0.005	4	1	25
0.006	4	1	25
0.007	4	2	50

During this study, the behaviour of the fish in the control was normal while the fish introduced into different concentrates of the herbicides shows different abnormal behaviour. These behaviours which include erratic swimming, restlessness, etc. were observed in fish exposed to the chemical as shown in Table 2. However, the behaviours became intense with increase in concentration. At higher concentration of 0.007 ml/l, the fish become very weak and settled at the bottom of the plastic tank. Normal colour and behavioural response was observed in the control experiment.

**Table 2:** Behavioural changes of *Clarias gariepinus* juveniles exposed to different concentrations of glyphosate.

Interval (h)	Behaviour
0	Normal behavioural response
24	Swimming in an erratic manner
48	Gulping for air near water surface
72	Overturn while swimming
96	Became dull and eventually dies

The changes or variation of physiochemical parameters are presented in Table 3 which shows that these parameters decreases with increase in concentration of the herbicide.

**Table 3:** Physicochemical parameters of concentrations of glyphosate exposed to *Clarias gariepinus* juveniles.

Concentration (ml/l)	pH		DO (mg/l)	
	Initial	Final	Initial	Final
0.000	7.84	7.93	4.30	4.55
0.004	7.93	7.15	4.59	5.20
0.005	7.94	7.13	4.64	4.19
0.006	7.85	6.97	4.84	4.03
0.007	7.71	6.81	4.69	4.47

Furthermore, the LC_50_ value was obtained based on probit analysis (using Biostat Pro 5.9.8 2015) and was found to be 0.0072 ml/l for 96 h of exposure to the glyphosate herbicide.

## 4 Discussion

Mortality profile of the research represented in Table 1 shows that percentage mortality of *Clarias gariepinus* juvenile increases with increase in concentration of glyphosate which is consistent with observation from [2]. Similarly, less mortality was found at 0.004 ml/l concentration while higher mortality was found at 0.007 ml/l concentration of glyphosate.

However, with highest concentration of glyphosate, various behavioural changes occur such as erratic swimming, gulping of air, loss of equilibrium and resting motionless at the bottom of the aquaria were observed (see Table 2), which was similar to observations made by Okayi et al. [5]. The erratic swimming, restlessness, gulping of air and resting motionless at the bottom of aquaria observed in the investigation are not only as a result of impaired metabolism but could be due to nervous disorder. This was because the behavioural changes were unnoticed before the application of glyphosate. This was also in line with previous studies [10], [11], [5]. Low concentration of glyphosate herbicide (0.004 ml/l) did not produce any serious change in the fish behaviour within 24 h. Meanwhile, higher concentration (0.007 ml/l) at 96 h showed loss of response to stimulus and death. This is proportional to increase in glyphosate herbicide concentration and duration of exposure. Hence, mortality is dose dependent [5].

The introduction of a toxicant into aquatic system might reduce DO value. This might be the reason why the fishes were stressed progressively with time before death. From the result obtained pH decreases with increase in concentration. DO also decreases with increase in concentration. Death recorded could therefore have occurred by this reason. This coincide with the result of a study on toxicity of dizensate (glyphosate herbicide) on *Clarias gariepinus* fingerlings [10].

Water quality plays a great role in influencing fish survival, reproduction and growth performance. They affect aquatic organisms by causing mortality and change in behaviour among species as a result of influences such as reduction in reproductive roles or alternations in competitive ability [10]. Physicochemical parameters such as DO and pH undergo a significant change resulting from increase in concentration of toxicant as shown in Table 3. This observation is consistent with Ayoola [11] who investigated the histopathological effects of glyphosate on *Clarias gariepinus* juveniles.

The LC_50_ value obtained showed that *Clarias gariepinus* is sensitive to glyphosate herbicide. The LC_50_ at 96 h is 0.0072 ml/l. Varying LC_50_ values have been obtained from different investigation on exposure of the same organisms to different herbicides. The differences in the values may be as a result of variation in the nature of herbicide, age of organism and environmental condition [12].

Statistical analysis showed that there was significant difference between the initial and final pH values (*p *< 0.05). However, there was no significant difference between the initial and final DO values (*p *> 0.05). Similarly, correlation analysis showed no significant relationship between DO and pH (*p* > 0.05).

## 5 Conclusion

The present investigation shows that mortality increases with increase in concentration of glyphosate herbicide which has manifested in behavioural changes. DO and pH were also affected as a result of the increase in the concentration. Hence, the higher the concentration the higher the mortality rate and change in behaviour, DO, and pH values. Conversely, the lower the concentration the less the mortality rate, change in behaviour, DO, and pH values. Therefore, glyphosate can induce mortality in *Clarias gariepinus* juveniles.

## Recommendations

1. The use of glyphosate near aquatic environment should be monitored to ensure that farmers use appropriate concentration that will not kill non-target organisms.
2. Biological methods should be used as an alternative to chemical methods of controlling weeds.
3. Government and other relevant bodies should enlighten farmers and public on the implication of herbicide to aquatic biota.

## Transparency document

Transparency Document
